# Meaningful Secret Image Sharing with Saliency Detection

**DOI:** 10.3390/e24030340

**Published:** 2022-02-26

**Authors:** Jingwen Cheng, Xuehu Yan, Lintao Liu, Yue Jiang, Xuan Wang

**Affiliations:** 1 College of Electronic Engineering, National University of Defense Technology, Hefei 230037, China; chengjingwen87@nudt.edu.cn (J.C.); liuta1989@163.com (L.L.); jiangyue17@nudt.edu.cn (Y.J.); wangxuan21d@nudt.edu.cn (X.W.); 2 Anhui Province Key Laboratory of Cyberspace Security Situation Awareness and Evaluation, Hefei 230037, China

**Keywords:** secret image sharing, random elements utilization model, statistical correlation, saliency detection, meaningful shadows, polynomial-based SIS

## Abstract

Secret image sharing (SIS), as one of the applications of information theory in information security protection, has been widely used in many areas, such as blockchain, identity authentication and distributed cloud storage. In traditional secret image sharing schemes, noise-like shadows introduce difficulties into shadow management and increase the risk of attacks. Meaningful secret image sharing is thus proposed to solve these problems. Previous meaningful SIS schemes have employed steganography to hide shares into cover images, and their covers are always binary images. These schemes usually include pixel expansion and low visual quality shadows. To improve the shadow quality, we design a meaningful secret image sharing scheme with saliency detection. Saliency detection is used to determine the salient regions of cover images. In our proposed scheme, we improve the quality of salient regions that are sensitive to the human vision system. In this way, we obtain meaningful shadows with better visual quality. Experiment results and comparisons demonstrate the effectiveness of our proposed scheme.

## 1. Introduction

With the development of artificial intelligence and internet technology, many studies have focused on the information security. Large amounts of images are transmitted in the cloud networks every day. It is worth paying attention to the transmission safety of sensitive images such as remote-sensing images and military images. Image encryption [1] and information hiding [2,3] are traditional image protection technologies, but they are not applicable in some scenarios. Secret image sharin g (SIS), as one of the applications of information theory in information security protection, has been widely used in many areas, such as blockchain [4], identity authentication [5,6] and distributed cloud storage [7,8].

In a secret image sharing scheme with a (k,n) threshold [9], a secret image is divided into *n* shadows and sent to *n* participants. If *k* or more shadows are collected, the original secret can be reconstructed. In contrast, less than *k* shadows reveal nothing of the secret image.

Generally, there are three main branches in SIS: visual cryptography (VC) [10,11], Chinese Remainder Theorem (CRT)-based SIS [12,13,14] and polynomial-based SIS (PSIS) [15,16,17,18]. PSIS is always adopted because of the lossless recovery, no pixel expansion and good visual quality shadows. The primitive polynomial can be used to realize PSIS, and Lagrange interpolation is exploited to reconstruct the original secret image. The polynomial multiplication is required as the main operation in Lagrange interpolation with a higher computational cost than VC and CRT-SIS. The Number Theoretic Transform (NTT) can be used to improve the performance of polynomial multiplication [19,20,21]. Thus, the recovery efficiency is improved, especially with large polynomial degrees. According to the characteristics of images, the existing PSIS is more commonly implemented over the integer field with a prime P, which is illustrated in Section 2.1.

In traditional SIS, the shadows are usually noise-like. These noise-like shadows easily attract the attention of attackers. On the other hand, it is difficult to distinguish noise-like shadows, which presents difficulties in shadow management. To facilitate shadow management and ensure transmission security, some researchers have committed to generating meaningful shadows. Meaningful SIS was first proposed by Ateniese et al. [22]. They applied visual cryptography to generate meaningful binary shadows. According to different design concepts, meaningful SIS can be classified into two categories.

The first design concept combines SIS with information hiding schemes. First, traditional SIS schemes are employed to share the secret image and obtain noise-like shadows. Then, an information hiding scheme is applied to embed noise-like shadows into cover images to make shadows meaningful. In the recovery phase, the noise-like shadows are first extracted from the covers, and the original secret image can be reconstructed by the recovery algorithm.

Yuan et al. [23] applied multi-cover adaptive steganography to share natural images. The secret image is adaptively embedded into the textured regions of cover images, but pixel expansion occurs in their scheme. Cheng et al. [24] employed a Gray code to obtain meaningful shadows. In their method, AMBTC compression is used to reduce the transmission bit rate. Chiu et al. [25] presented a (2,n) threshold progressive visual cryptography scheme to generate meaningful shadows. He et al. [16] used LOCO-I compression to reduce the statistical correlations between neighboring pixels and obtain meaningful shadows based on steganography. Derya et al. [26] introduced a method to generate meaningful shares with Arabic letters. They embedded shares into the R, G and B channels of their RGB cover images with steganography.

All the abovementioned schemes are based on an information hiding scheme to generate meaningful shadow images. Shadows in these schemes possess some information hiding properties, such as steganography resistance. However, limited by the embedding rate of information hiding schemes, these sharing schemes must minimize the size of the secret image. To obtain better visual quality, they usually have pixel expansion.

The second design concept does not require steganography. With the constraints of secret pixel values and cover pixel values, researchers have improved the sharing algorithm to generate a shadow pixel, which is close to its corresponding cover pixel. As a result, the shadows are meaningful and similar to the cover images.

Wu et al. [27] constructed meaningful shadows based on random grid visual cryptography. Their covers consisted of binary images. Yang et al. [28] applied digital half-toning technology to improve the visual quality of meaningful binary shadow images. Liu et al. [29] utilized a sharing map and a sharing pool to obtain meaningful shadows. They made the most significant bit of each shadow equal to the higher bit of the corresponding cover image. Yan et al. [30] presented a CRT-based SIS that can generate meaningful shadows. A modular operation is applied to share the secret image. They also used binary images as covers.

These methods mostly use binary cover images, which have lower visual quality than grayscale images. Some of them only use one cover image; thus, their shadows are all similar to the cover with the same content. Generally, neither of these two kinds of schemes has high visual quality, and the secret images are shared as pure data. However, as natural images, adjacent pixels in a cover image have a strong correlation in color, texture and luminance, which are not considered in the above two kinds of schemes.

The motivation of this article is to propose a meaningful SIS scheme with saliency detection to improve the visual quality of shadows. Since the salient regions are quite different from adjacent regions in color, texture, or luminance, human attention always focuses on an image’s salient regions. In the proposed scheme, we try to improve the visual quality of salient regions in shadows; then, the overall visual quality can be improved apparently. LC algorithm is utilized to identify the salient regions, and a random elements utilization model is exploited to screen shared values and distribute more identical bits to salient regions. In this way, salient regions in shadows are more similar to the same salient regions in covers. The experiment results show that our shadows have better visual quality than the relative schemes.

We organize this article as follows: Section 2 introduces the principle of polynomial-based SIS and a saliency detection method named LC. The proposed scheme is presented in Section 3. Experiments and comparisons with relative schemes are presented in Section 4. Section 5 concludes this paper.

## 2. Preliminaries

### 2.1. Polynomial-Based Sis

In (k,n) threshold polynomial-based SIS, as seen in Equation (1), a (k−1) degree polynomial is constructed in a finite field GF(P), where *P* is a prime number. In the sharing phase, we set the secret pixel value $s=a_{0}$ and randomly select the coefficients $a_{1}$, $a_{2}$, ⋯, and $a_{k-1}$ within the interval $\left[0,P\right)$. f(i) is calculated as the share value according to Equation (1). After all secret pixel values have been shared, *n* shadows can be obtained.

(1) $$f\left(x\right)=\left(a_{0}+a_{1}x+\cdots +a_{k-1}x^{k-1}\right)\;\mathrm{mod}\;P$$

As described in Equation (2), Lagrange interpolation is used to reconstruct the original polynomial if *k* or more shares are gathered. In this way, *k* coefficients are calculated, and $a_{0}$ is the recovered secret pixel value $s{}^{\prime }$. The polynomial cannot be reconstructed with fewer than *k* shares; consequently, no secret information can be revealed.

(2) f(x)=∑i=1kf(xi)∏j=1j≠ik(x−xj)(xi−xj)

### 2.2. Saliency Detection

The human vision system can quickly determine the interesting regions in a complex image [31]. The salient regions are more likely to attract the attention of the human eyes because they are quite different from other regions in terms of texture, color and luminance. Saliency detection is used to simulate the human vision system to obtain the salient regions in an image.

Here, we introduce a pixel-level saliency detection algorithm based on a pixel’s contrast to all other pixels (the so-called LC algorithm). The LC algorithm is proposed by Zhai and Shah [32], and it is one of the state-of-the-art traditional methods for saliency detection.

The LC algorithm builds a saliency map with a color contrast between pixel values. The saliency value of pixel $P_{t}$ is defined as Equation (4), which equals the sum of Euclidean distances between the pixel value of $P_{t}$ and all the other pixels of image *I*.

(3) $$Sal\left(P_{t}\right)=\sum_{\forall P_{i}\in I}∥P_{t}-P_{i}∥$$

The LC algorithm is suitable for grayscale images because the saliency value of pixel $P_{t}$ is the sum of Euclidean distance between pixel $P_{t}$ and all the other pixels in the image. The saliency value computation for a pixel $P_{t}$ can be optimized with the use of image color histograms as:

(4) $$Sal\left(P_{t}\right)=\sum_{n=0}^{255}f_{n}D\left(t,n\right)$$

where *t* is the color value of pixel $P_{t}$; D(t,n) is the color difierence between $P_{t}$ and $P_{n}$; $f_{n}$ is the probability of pixel value *n* in image *I*.

**Figure 1:**
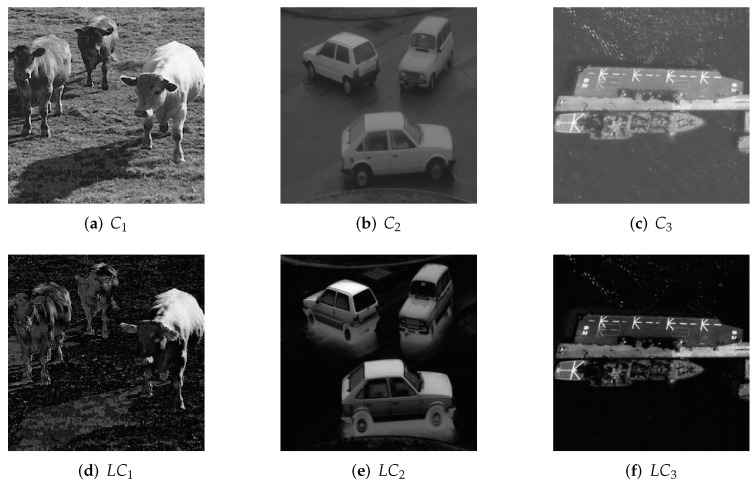
LC algorithm results. (**a**–**c**) original grayscale images; (**d**–**f**) saliency maps for original images.

Figure 1 illustrates our experiment images their saliency maps with LC algorithm. Compared with other saliency detection methods [33,34,35], such as AC [36], FT [37], CA [38], RC [33], LC takes a little running time because LC is purely computational with low computational complexity. According to Figure 1 and the comparing results illustrated in [33], the precision of LC is satisfied, and the saliency maps with LC algorithm are accordant with human eye perception. However, other traditional saliency detection like FT [37], AC [36] and RC [33] can also be used in our scheme after some adjustments since they are designed for color images.

Recent saliency detection researches are focused on Convolutional Neural Networks (CNN). The CNN-based saliency detection methods may obtain more precise saliency maps. However, the CNN models are complex, which leads to high computation complexity and long running time. Moreover, the LC algorithm is good enough for our scheme as demonstrated in our experiment results.

## 3. The Proposed Scheme

Here, we introduce our proposed scheme. First, the concept of our method is presented. Then, the details of our sharing method are described in Algorithm 1.
**Algorithm 1.** The Sharing Phase of Our Proposed Scheme.**Input**: a grayscale secret image *S* with a size of *W × H*; *n* grayscale cover images $C_{i}$ with a size of *W × H*; **Output**: *n* meaningful shadows $SC_{1}$, $SC_{2}$, …,$SC_{n}$.**Step 1:** Use the LC algorithm to calculate the saliency values for every pixel in cover $C_{i}$. Note the saliency values as $Sal_{i}^{1}$, $Sal_{i}^{2}$, …, $Sal_{i}^{W\times H}$. **Step 2:** Compare $Sal_{1}^{t}$ and $Sal_{2}^{t}$, …,$Sal_{n}^{t}$, which are the saliency values of the same pixel position $P_{t}$ of *n* covers..**Step 3:** Utilize the random elements utilization model and the result of Step 2 to screen the shadow pixel values.**Step 4:** Repeat Step 2 and Step 3 until all secret image pixels have been shared.**Step 5:** Output *n* meaningful grayscale shadow images $SC_{1},SC_{2},\cdots SC_{n}$.

### 3.1. Design Concept

**Figure 2:**
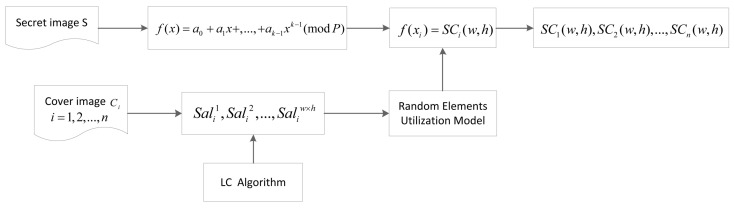
Design concept of the proposed scheme.

The design concept of our scheme is illustrated in Figure 2. In our method, the LC algorithm is utilized to calculate the saliency values (noted as $Sal_{i}^{1}$, $Sal_{i}^{2}$, …, $Sal_{i}^{W\times H}$) for every pixel of each cover image. The saliency value indicates the pixel’s significance in the image. A larger saliency value means a more significant role that the pixel plays in the image. The saliency maps of covers can be obtained through the LC algorithm.

To generate meaningful shadows, we set some specific conditions during the sharing phase. In terms of visual quality, the higher bits of a pixel value are more important than the lower bits. If we keep more higher bits identical for corresponding pixels, the two images are more similar. However, there is a limit to the sum of identical bits, and for *n* different cover images, the salient regions are different. If we distribute more identical bits to salient regions, the shadow quality will be better.

The design concept of our scheme distributes more identical bits to salient regions according to the saliency values. A random elements utilization model is used to screen the shadow pixels that satisfy these specific conditions. In this way, the salient regions in shadows are more similar to the same regions of the corresponding cover image. Therefore, the shadows will obtain better visual quality.

### 3.2. Random Elements Utilization Model

According to the principle of PSIS, coefficients $a_{i}$(1≤i≤k−1) are selected randomly to gain shared values. Different $a_{i}$ lead to different shared values, and the coefficients $a_{i}$ are regarded as the random elements in the sharing phase.

The random elements utilization model is exploited to screen shared values to obtain meaningful shadows with better quality. The sum of identical bits is expected as more as possible to obtain better visual quality, *t*. Since $w_{i}$ is noted as the identical higher bits that distribute to $SC_{i}$, we correlate the random elements utilization model with a maximize problem as follows.

(5) $$Maximize\sum_{i=1}^{n}w_{i}$$

(6) $$s.t.\left\{\begin{array}{c} \sum_{i=1}^{n}w_{i}\leq 8\left(k-1\right)\\ f\left(x_{t}\right)=s+\sum_{m=1}^{k-1}a_{m}x_{t}^{m}\;\mathrm{mod}\;P\\ a_{m}\in Z,a_{m}\in \left(0,P\right]\\ s\in \left[0,255\right]\\ f\left(x_{t}\right)\in \left[0,255\right]\\ t=1,2,...,n \end{array}\right.$$

The maximization problem can be solved by the integer linear programming technique. According to the random elements utilization model, identical higher bits $w_{i}$ are distributed to $SC_{i}$, respectively. The shared values in $SC_{i}$ can be screened while keeping $w_{i}$ identical bits with the corresponding cover pixels; thus, the shadows are meaningful and similar to the corresponding covers.

### 3.3. Our Scheme

The detailed sharing steps are described in Algorithm 1, and we make the following points:

1. The salient regions have a greater influence on human visual perception than other regions. We improve the visual quality of the shadows by improving the visual quality of the salient region.
2. We apply the LC algorithm to calculate the saliency values for every pixel in each cover. Saliency values are used to measure the importance of corresponding pixels. A larger saliency value indicates that the cover pixel is in a salient region, while a cover pixel with a smaller saliency value is in a non-salient (less important) region.
3. In our scheme, the sum of identical higher bits for all shadows is limited. With the random elements utilization model and the comparison results in Step 2, we distribute more identical higher bits to salient regions and less to non-salient regions. Thus, the salient regions obtain better visual quality and are more similar to corresponding regions in cover images. Moreover the distribution process is adaptive to different shadow images.
4. There is a limitation on the sum of identical higher bits for all shadows. Since we choose 257 as the prime number, the total number of sharing values is $257^{k-1}\approx 2^{8(k-1)}$. In our scheme, the total number of satisfied sharing values is $2^{\sum_{i=1}^{n}w_{i}}$. To ensure the successful sharing process, the sum of identical higher bits should be subject to $\sum_{i=1}^{n}w_{i}\leq 8\left(k-1\right)$.
5. Polynomial-based SIS is used to share the secret pixels, and a prime number *P* of 257 is chosen to ensure lossless recovery. In the recovery phase, the secret image can be losslessly reconstructed by Lagrange interpolation. The recovery operation complexity is $O(klog^{2}k)$ [39].

## 4. Experiments and Discussion

In this section, we first exhibit our experimental results. Then, comparisons with relative schemes are performed to show the effectiveness of our proposed scheme with the same threshold and secret image. In addition, a discussion is provided.

### 4.1. Image Illustration

**Figure 3:**
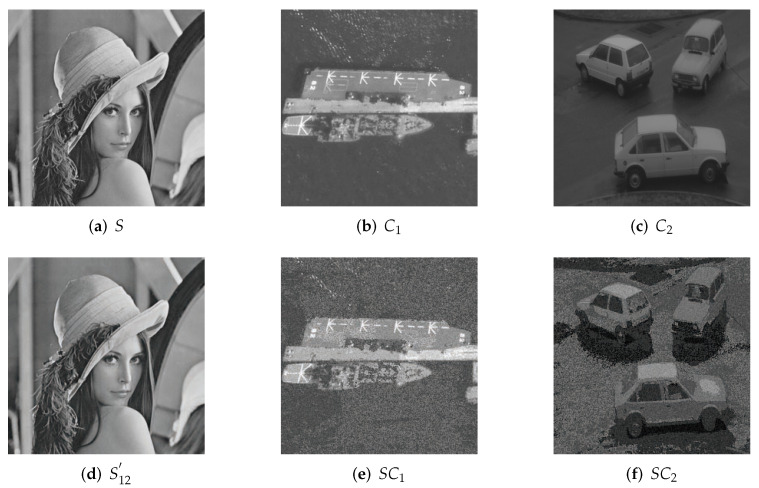
(2,2) threshold experimental results of our proposed method; (**a**) grayscale secret image; (**b**,**c**) two grayscale cover images; (**d**) recovered secret image with two shares; (**e**,**f**) two meaningful shadow images.

The experimental results of our proposed scheme with the (2,2) threshold are exhibited in Figure 3; Figure 3a shows the grayscale secret image. Two grayscale cover images are shown in Figure 3b,c; the recovered secret image is illustrated in Figure 3d; Figure 3e,f demonstrates two shares. The shares are not noise-like but meaningful. They are similar to the corresponding cover images. The details of the shadows can also be accurately recognized. For example, as Figure 3e illustrates, the lines on the deck of the warship can be recognized easily and accurately. All the shares and reconstructed secret images have the same size as the original secret image, and no pixel expansion occurs.

### 4.2. Quality Evaluation Metrics

Our experiment evaluates the image quality with the statistical correlation between shadows and corresponding covers. Here, three statistic-based metrics are introduced to obtain the statistical correlation between two images. In SIS, peak signal-to-noise-ratio (PSNR), structural similarity (SSIM) [40], and universal quality index (UQI) [41] are widely used statistic-based metrics.

PSNR between image S and S’ is calculated as Equation (7).

(7) $$PSNR=10\log_{10}\frac{255^{2}}{MSE}\left(dB\right)$$

(8) $$MSE=\frac{1}{W\times H}\sum_{i=1}^{W}\sum_{j=1}^{H}{\left[S^{\prime }\left(i,j\right)-S\left(i,j\right)\right]}^{2}$$

where MSE represents the mean square error of image S’ and image S. The value range of PSNR is $\left[0,+\infty \right]$. The larger the value of PSNR is, the more similar the two images are.

Different from PSNR, SSIM evaluates image similarity from brightness, contrast, and structure. SSIM is defined as Equation (9).

(9) $$SSIM\left(x,y\right)={\left[l\left(x,y\right)\right]}^{\alpha }\cdot {\left[c\left(x,y\right)\right]}^{\beta }\cdot {\left[s\left(x,y\right)\right]}^{\gamma }$$

where

(10) $$\begin{array}{c} l\left(x,y\right)=\frac{2\mu_{x}\mu_{y}+C_{1}}{{\mu_{x}}^{2}{\mu_{y}}^{2}+C_{1}}\\ c\left(x,y\right)=\frac{2\sigma_{x}\sigma_{y}+C_{2}}{{\sigma_{x}}^{2}{\sigma_{y}}^{2}+C_{2}}\\ s\left(x,y\right)=\frac{2\sigma_{xy}+C_{3}}{\sigma_{x}\sigma_{y}+C_{3}} \end{array}$$

UQI can also be used to evaluate image distortion, and its value range is $\left[-1,1\right]$. The larger value of UQI indicates less distortion and better quality. UQI is calculated as follows.

(11) $$UQI=\frac{4\mu_{x}\mu_{y}\sigma_{xy}}{\left(\mu_{x}^{2}+\mu_{y}^{2}\right)\left(\sigma_{x}^{2}+\sigma_{y}^{2}\right)}$$

The three statistic-based metrics can be directly used for grayscale images. In the comparison experiment, the input binary images are first grayed, and the pixel value is multiplied by P−1. Then, the three metrics can be used to evaluate the image quality.

### 4.3. Comparisons with Relative Methods

In this section, we compare the proposed scheme with two relevant meaningful SIS methods: Liu et al. [29] and Yan et al. [30]. These methods both obtain meaningful shadows. To better show the advantages of our proposed scheme, we use the same secret image and thresholds in the comparative experiments.

**Figure 4:**
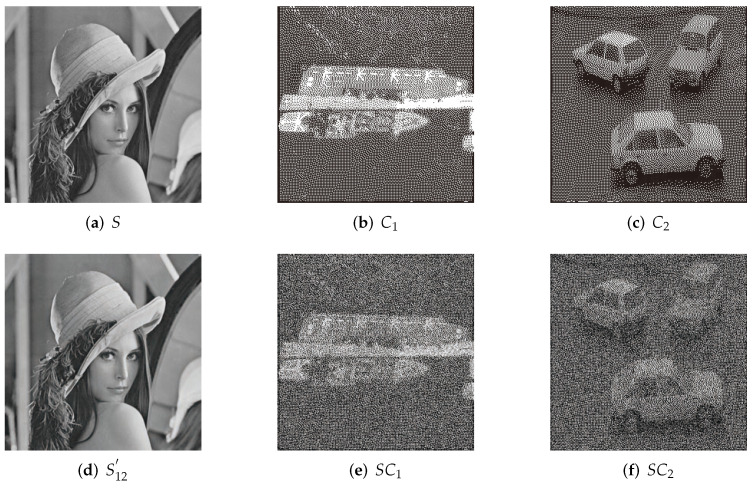
(2,2) threshold experimental results of Liu et al.; (**a**) grayscale secret image; (**b**,**c**) binary cover images; (**d**) recovered secret image; (**e**,**f**) meaningful shadow images.

Liu et al. [29] obtained meaningful shadows by employing a sharing map and sharing pool. The sharing pool is determined by the secret pixel values and cover pixel values. Different binary images are used in their method as covers. They choose appropriate shared pixel values from the sharing pool to obtain meaningful shadows. We use two binary covers that have the same content as our covers to realize their (2,2) threshold experiment. The results are illustrated in Figure 4.

**Figure 5:**
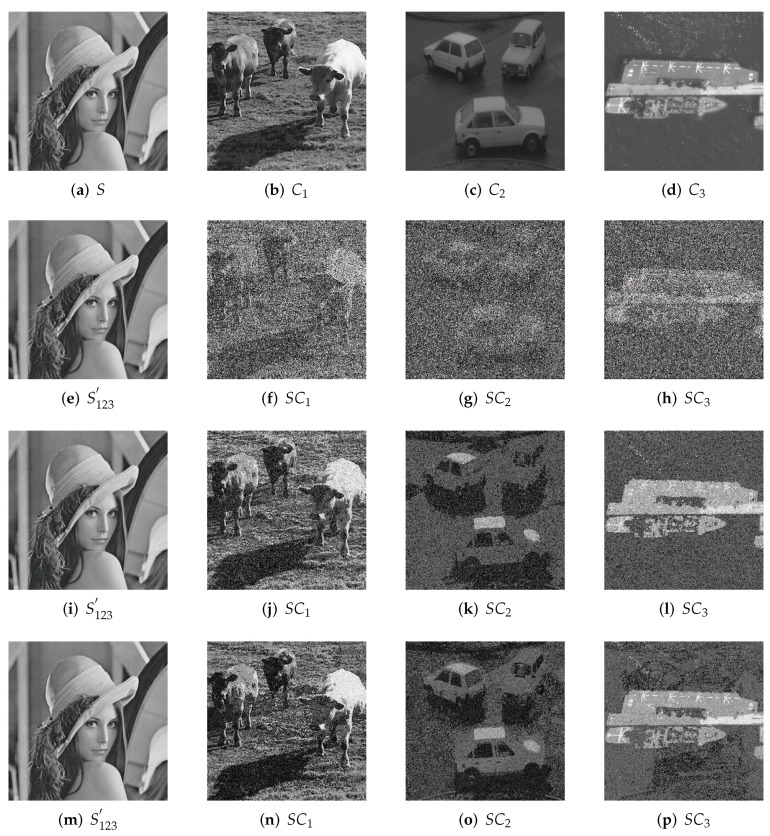
(2,3) threshold experimental results of IBDR, Yan et al. and our proposed method; (**a**) grayscale secret image; (**b**–**d**) three grayscale cover images; (**e**–**h**) results of Yan et al.’s scheme; (**i**–**l**) results of IBDR scheme; (**m**–**p**) results of our proposed scheme.

Yan et al. [30] presented a meaningful SIS scheme based on the Chinese Remainder Theorem. They also utilized binary images as covers, and the secret image was shared by a modular operation. We also realize their scheme with a (2,3) threshold. The results are exhibited in Figure 5e–h.

To show the effectiveness of saliency detection in our scheme, we designed a comparison experiment without saliency detection. In the comparison experiment, the threshold and the sum of the identical bits are equal our proposed scheme. We removed the saliency detection, and identical bits were distributed randomly among each shadow. The comparison experiment we refer to as IBDR. Figure 5i–l shows the (2,3) threshold results of the IBDR scheme.

The results of our proposed scheme with the (2,3) threshold are illustrated in Figure 5m–p. Compared with our experimental results with relative schemes in Figure 4 and Figure 5, we can see that the visual quality of the shadows in our scheme is obviously better than Liu et al. [29] and Yan et al. [30]. In their shadows, only the outlines of the objects can be distinguished. In the IBDR scheme, the visual quality is higher than Liu et al. and Yan et al., but it failed to display some of the details of the shadows. For example, in Figure 5l, we can identify that it is a warship, but we cannot determine the lines on the deck, and the details of the ship cannot be recognized clearly. In contrast, in the results of our proposed scheme, the lines on the deck of the warship are accurately illustrated in Figure 5p. Moreover, as shown in Figure 5k,o, the outline of the cars in our scheme is clearer than that of the IDBR scheme.

The visual quality of images can be measured by PSNR, SSIM and UQI. Table 1 and Table 2 exhibit the statistical results of our proposed scheme and the comparison schemes. Compared with Liu et al. [29] and Yan et al. [30], the visual quality is improved significantly. This is also demonstrated by the PSNR values in Table 1, but the PSNR values of IBDR are close to our scheme. For further analysis, we calculated SSIM and UQI for these two schemes, as shown in Table 2. The statistical data show that our method performs better in SSIM and UQI.

### 4.4. Analysis and Discussion

According to Figure 4 and Figure 5 and Table 1 and Table 2, some analyses are given as follows.

1. Our scheme significantly improved visual quality compared with Liu et al. [29] and Yan et al. [30].
2. The PSNR of the IBDR method is close to ours. However, PSNR is calculated based on the discrepancy between the corresponding two pixel values, while the visual characteristics of human eyes are not taken into account. For example, human eyes are sensitive to luminance and texture and are usually influenced by the neighboring regions around the target object. The PSNR values are often inconsistent with the subjective judgment of human eye perception.
3. To further compare our scheme with IBDR, we calculated the indicators SSIM and UQI, which can better reflect the overall structure of images. As exhibited in Table 2, the higher values of SSIM and UQI show that our scheme is more effective than IBDR.
4. In our scheme, saliency detection is applied, which can effectively improve the visual quality of salient regions in shadows. For instance, the lines on the deck of the warship in Figure 3e can be clearly distinguished, but they are blurred in the corresponding shadows of other relative schemes. Our scheme exhibits the details of shadow images more accurately. The structural characteristics are used in saliency detection, so the outline of the cars in Figure 5o are clearer than in Figure 5k. These are also demonstrated with SSIM and UQI in Table 2.
5. The relative meaningful SIS schemes process each pixel individually. However, the color, texture and luminance among neighboring pixels have a strong correlation. They are sensitive to human eye perception. Our proposed scheme takes the correlation among neighboring pixels and structural characteristics into account by utilizing saliency detection. According to the random elements utilization model, the identical higher bit distribution process is adaptive to different shadow images. Then, the visual quality of saliency regions of shadows can be improved adaptively.
6. Our scheme performs well with small thresholds such as (2,2) and (2,3). For larger thresholds such as (4,4) or (4,5), the total number of identical bits is 8(k−1)=24. Because there are enough identical higher bits for each pixel and the lower bits have a smaller influence on visual quality, both the salient and less salient regions can obtain satisfied visual quality. In this condition, saliency detection is not very effective with large thresholds.
7. The LC algorithm can identify the salient regions accurately in our scheme. However, there are also some limitations. The sum of Euclidean distances between pixel values is calculated to obtain the saliency map in the LC algorithm. Mistakes will be involved when pixels with rare pixel values mistakenly gain high saliency values. Other saliency detection methods, such as FT [37], AC [36] and RC [33], can also be used in our scheme to obtain accurate saliency maps.

## 5. Conclusions

In this article, we design an SIS scheme with saliency detection to obtain meaningful shadows. Saliency detection methods such as the LC algorithm are used to determine the salient regions, which are sensitive to the human vision system. In this way, the shadows in our scheme have better visual quality than the relative method. The experimental results indicate the effectiveness of our scheme. In addition, our future work will focus on meaningful SIS for color images with other saliency detection methods.

## Figures and Tables

**Table 1:** PSNR comparison between the proposed scheme and relative schemes.

Threshold	Schemes	Shadows1	Shadow2	Shadow3	Average
(2,2)	Liu	10.6781	10.6942		10.6861
	Ours	22.4381	20.4256		21.4318
(2,3)	Yan	7.9441	8.2266	8.1357	8.1021
	IBDR	16.4911	17.2531	17.5863	17.1101
	Ours	16.7203	18.2884	18.2662	17.7583

**Table 2:** SSIM and UQI comparison between our proposed scheme and BIDR scheme.

Schemes	Metrics	Shadows1	Shadow2	Shadow3	Average
IBDR	SSIM	0.4362	0.1089	0.1677	0.2376
	UQI	0.4659	0.0751	0.1461	0.2291
Ours	SSIM	0.4647	0.1599	0.2213	0.2817
	UQI	0.4892	0.1182	0.1853	0.2642

## Data Availability

Not applicable.
